# Seismic attenuation transients reveal progressive crustal modification before and during the 2023 Türkiye earthquakes

**DOI:** 10.1038/s41598-026-52463-1

**Published:** 2026-05-15

**Authors:** Simona Gabrielli, Aybige Akinci, Yijian Zhou, Edoardo Del Pezzo, Luca De Siena

**Affiliations:** 1 Istituto Nazionale di Geofisica e Vulcanologia, Rome, Italy; 2 Caltech GPS, Pasadena, CA US; 3 Osservatorio Vesuviano, Istituto Nazionale di Geofisica e Vulcanologia, Napoli, Italy; 4 Istituto Andaluz de Geofisica, Universidad de Granada, Granada, Spain; 5 Dipartimento di Fisica e Astronomia “Augusto Righi”, Alma Mater Studiorum, 12, Bologna, Italy

**Keywords:** Natural hazards, Solid Earth sciences

## Abstract

We present the first spatiotemporally resolved separation of scattering and intrinsic seismic attenuation across the East Anatolian Fault Zone, capturing their evolution before and during the 2023 Türkiye earthquake doublet. Our results reveal a fault-segment–dependent decoupling between scattering and intrinsic absorption, indicating that the two components are governed by distinct physical processes rather than a single attenuation mechanism. Scattering attenuation intensified along the Çardak-Sürgü rupture, delineating spatial variations associated with strong rheological contrasts, whereas intrinsic attenuation exhibits broader temporal variability. Time-series analysis identifies measurable attenuation changes, reaching up to ~ 40%, associated not only with the mainshocks but also with moderate pre-sequence seismicity. These observations indicate that seismic attenuation records a temporal fingerprint of evolving crustal conditions. Overall, our findings demonstrate that resolving attenuation components provides diagnostic capability by capturing crustal modifications beyond the general post-earthquake damage observation.

**Supplementary Information:**

The online version contains supplementary material available at 10.1038/s41598-026-52463-1.

## Introduction

The Arabia–Eurasia collision zone in eastern Anatolia provides a key example of regionally distributed deformation, characterized by microplate formation and widespread lithospheric reorganization. The Anatolian microplate is defined as a relatively rigid block, bounded to the north by the North Anatolian Fault Zone (NAFZ) and to the east and southeast by the East Anatolian Fault Zone (EAFZ), two major transform fault systems that accommodate the westward tectonic extrusion of Anatolia from the Arabia–Eurasia collision front^1–3^.

This tectonic extension is driven by the northward convergence of the Arabian Plate with the Eurasian Plate at a rate of ~ 20–25 mm/yr^4^ and is manifested in the high seismicity and complex deformation observed in eastern Türkiye. The EAFZ, in particular, accommodates the westward motion of Anatolia relative to Arabia and has evolved into a major intracontinental strike-slip fault. It is seismically active and has hosted numerous large historical and instrumental earthquakes, including the 2020 Elazığ (Mw 6.8).

On 6 February 2023, a Mw 7.8 earthquake ruptured the EAFZ in southern Türkiye^5–7^. It was followed nine hours later by the Mw 7.5 Elbistan earthquake and, twenty days after the doublet, by the Mw 6.4 Antakya aftershock. A wide range of geophysical and geodetic studies has investigated various aspects of the 2023 sequence, including rupture geometry, slip distribution, ground motion patterns, and supershear rupture^5,8–10^. However, seismic attenuation, encompassing both intrinsic energy loss (absorption) and wavefield redistribution due to small-scale heterogeneities (scattering), remains relatively unexplored in the context of this sequence. Yet, it is a powerful diagnostic tool for assessing fault-zone damage, crustal properties, and rheological heterogeneity.

Seismic attenuation is highly sensitive to fracturing, fluid content, and lithological contrasts, making it especially effective for imaging active fault zones and monitoring their post-seismic evolution^11,12^. Its two components, scattering and absorption, have proven particularly responsive to the spatio-temporal evolution of seismic sequences along crustal fault systems^13–18^. Several studies have tracked temporal and spatial changes in attenuation^19,20^, revealing notable variations linked to fracture development, fluid migration, and rheological contrasts in fault zones.

Previous investigations of seismic attenuation in the broader region of Eastern Anatolia have reported high attenuation associated with partial melting, fractured lithologies, and fluid pathways in the crust^21–24^, but few have focused on how attenuation evolves during and after large earthquake sequences. Zor et al.^21^ found high to moderate Lg attenuation (Q_0_​ ~ 70–100) in the East Anatolian Plateau, likely due to intrinsic attenuation arising from recent tectono-volcanic activity. In contrast, beneath the Turkish plateau, Q_0_​ values between 100 and 200 were attributable to the combined effects of scattering and absorption^21^. Sertçelik^25^ calculated the Qc along the EAFZ, obtaining an average value of Q_c_ = 57.5f^0.82^, reflecting strong variability across the different regions of the fault system and its heterogeneities^25^. Izgi et al.^23^ investigated the attenuation of the Central Anatolian Plateau, highlighting high attenuation beneath Central Anatolia due to the presence of thick volcanic rocks^23^.

**Fig. 1:**
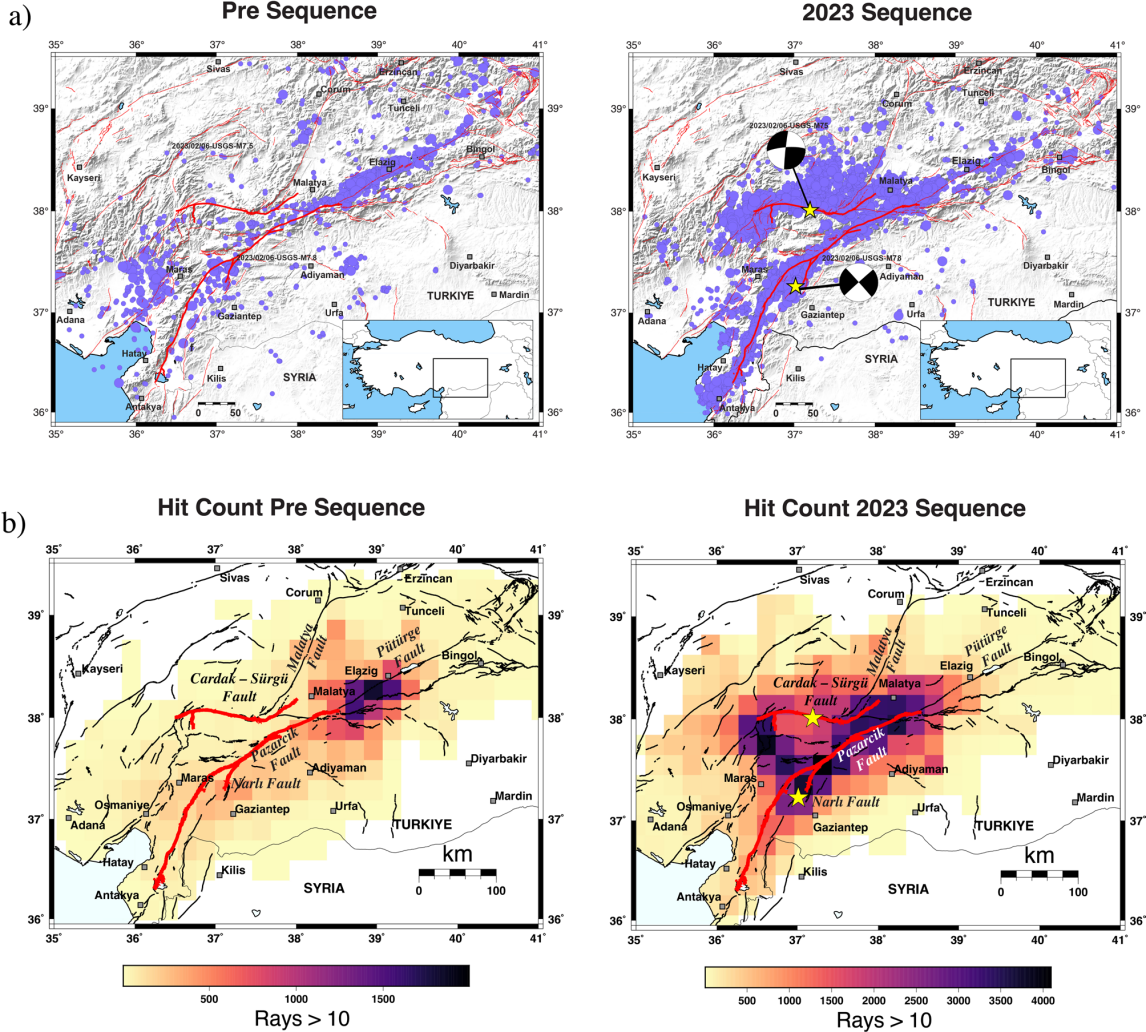
**a**) Events distribution for each dataset: Pre-sequence (left) and 2023 main sequence (right). ents that were activated during the 2023 Kahramanmaraş earthquake sequence. **b**) Hit Count map, showing the number of rays (> 10) passing through each cell for the two sequences. For both maps, yellow stars are the mainshocks of the 2023 seismic sequence. Thick red lines delineate the fault segments that were activated during the 2023 Kahramanmaraş earthquake sequence. Light red (**a**) and black lines (**b**) are the fault from the Mineral Research and Exploration (MTA) catalogue. Maps in a and b have been generated using GMT (v6.6.0, https://github.com/GenericMappingTools/gmt/releases).

In this study, we aim to map the spatial and temporal variation of scattering and intrinsic attenuation across the Eastern Anatolian region before and after the 2023 earthquake sequence. To achieve this, we analyse a comprehensive seismic dataset of over 125,000 waveforms, recorded by 64 broadband stations across the region (**Figure S1**). These data were processed using AI-PAL^26^, an automated workflow for phase picking, event association, relocation, and matched filtering of continuous seismic records, which integrates a rule-based detector PAL^27^ with a regionally trained deep-learning phase picker. The PAL detections, generated using STA/LTA and phase association algorithms, served as training labels for the SAR model, which was optimized using augmented waveform data from the KO and TU networks. We selected a quality-filtered subset of the catalogue requiring ≥ 10 associated stations per event, yielding 33,851 well-located earthquakes and 451,268 P-S phase pairs. This post hoc filtering ensures dense raypath coverage and robust resolution of attenuation parameters. Full methodological details are provided in Zhou et al.^26^. We selected all the waveforms having an SNR (signal-to-noise ratio) greater than 3 and events recorded by at least 10 stations. After this selection, the final number of waveforms is 20,722 for the Pre-Sequence and 105,318 for the Sequence. The datasets and their hit counts (ray density per cell) are represented in Fig. 1. It is important to note that in this dataset, magnitudes are computed using ML rather than Mw.

For the imaging, the analyses were performed at a central frequency (fc) of 1.5 Hz and 12 Hz (**Supplementary Material**). We focus on 1.5 Hz, as this frequency band allows us to detect the main tectonic structures and image features with linear dimensions of the order of ~ 2 km. Indeed, assuming an S-wave velocity of 3.5 km/s, the wavelength λ = v/f at 1.5 Hz is ~ 2.3 km. This frequency band was also used in our previous study in other tectonic areas (Gabrielli et al., 2022, 2023; Napolitano et al., 2023), with good agreement with independent tectonic studies. We further selected 12 Hz to investigate higher frequency anomalies. This allows us to verify the persistence of the main features at different scales.

The catalogue analysis for the attenuation techniques used in the paper is divided into two time periods:

1. January 2020–5th February 2023 (hereafter, Pre-Sequence phase).
2. 6th February − 31 st May 2023 (hereafter, Sequence phase).

We apply peak delay analysis to quantify scattering and use coda-wave modelling to estimate intrinsic attenuation (Qc^− 1^), both within a regionalization framework that enables mapping lateral changes in attenuation properties over time. By integrating these results with existing seismological observations, our study provides new constraints on the upper-crustal structure. It offers insights into fault-zone evolution and deformation processes in eastern Türkiye following large-magnitude seismic events.

## Results

### Spatial and temporal variability of peak delay time- scattering attenuation

**Fig. 2:**
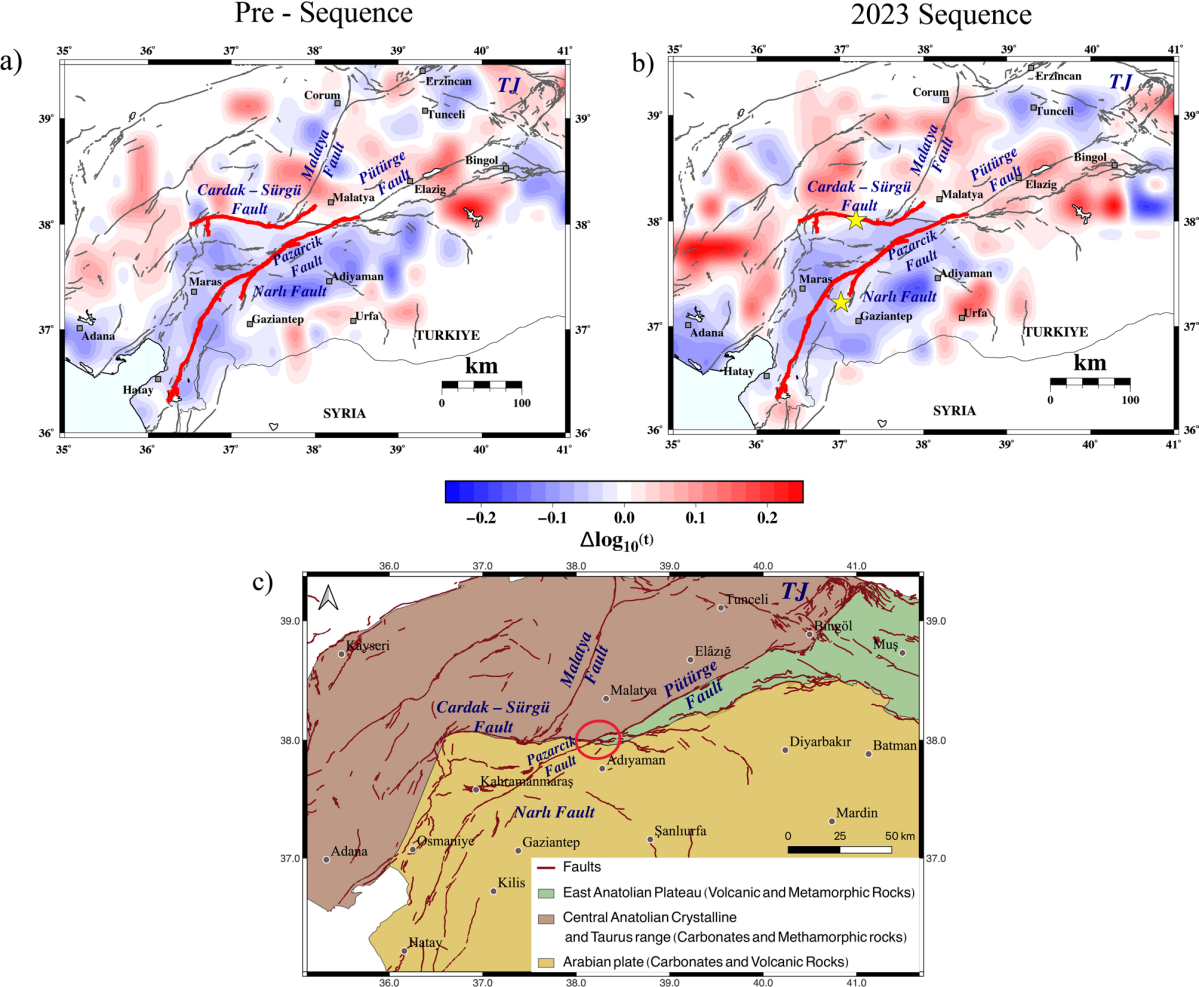
**a)** Spatial and temporal variation of peak delay at *fc* = 1.5 Hz during (a) the pre-seismic sequence (left) and the 2023 sequence (right). The maps display the absolute values of the frequency-dependent peak delay, Δlog₁₀t(*f*). Negative values (cold colours, blue) indicate low-scattering attenuation, while positive values (hot colours, red) correspond to high-scattering attenuation. The average Δlog₁₀t(*f*) over the region is approximately zero, serving as a reference. Only the blocks traversed by ten or more ray paths, as defined in Fig. 1b. Thick red lines delineate the fault segments that were activated during the 2023 Kahramanmaraş earthquake sequence, and yellow stars represent its mainshocks. **(b)** Geological framework of the area. (modified from Dilek and Sandvol^49^. The red circle represents a seismic gap on the EAFZ.

The comparison between the two seismic phases indicates temporal variations in scattering and common features, despite differences between the datasets, at both 1.5 Hz (Fig. 2) and at 12 Hz (**Figure S2**). Low scattering is constant below the Cardak-Sürgü fault till the area of the Pütürge fault before and during the 2023 sequence (Fig. 2a, b). Above the Cardak-Sürgü fault, however, in the pre-sequence (Fig. 2a), we observe a mix of low and high scattering, which becomes mainly high scattering during the sequence (Fig. 2b).

High scattering is observed around the Pütürge fault in Elâzığ, and in the area west of Maras and Osmaniye during both sequences (Fig. 2a, b).

The area between Elâzığ and Bingol is consistently characterized by high scattering, whereas Adana (southwest of the map) consistently exhibits low scattering.

The Amik basin (in the southern sector, Fig. 2b) during the sequence is characterized by high scattering, as it is the Triple Junction Area (**TJ**).

For all maps, grey (a, b) and red (c) lines are the faults from the Mineral Research and Exploration (MTA) catalogue. Main faults’ names are written in blue. Maps in a and b have been generated using GMT (v6.6.0, https://github.com/GenericMappingTools/gmt/releases), map in c using QGIS-LTR (v3.22.12-Białowieża, https://qgis.org/downloads-list/).

### Spatial and temporal variability of Coda Wave’s attenuation - absorption

**Fig. 3:**
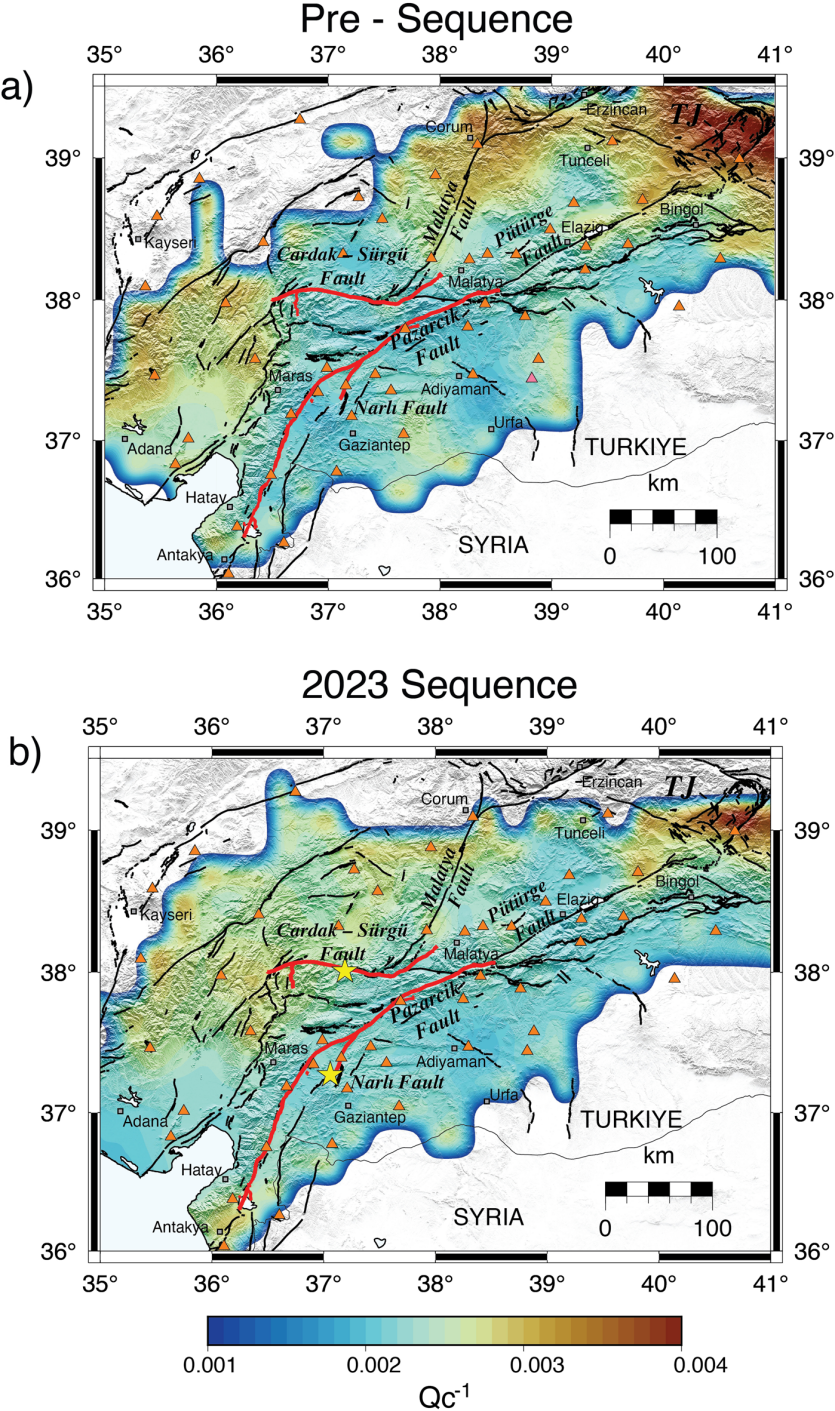
Spatial and temporal variation of the inverse coda quality factor (Qc⁻¹) at a central frequency of 1.5 Hz, shown for: **a**) the pre-seismic sequence and **b**) the 2023 main sequence. The maps display only grid cells traversed by at least five independent ray paths, ensuring statistical reliability of the Qc^− 1^ estimates. Thick red lines delineate the fault segments that were activated during the 2023 Kahramanmaraş earthquake sequence, and yellow stars represent its mainshocks. Black lines are the fault from the Mineral Research and Exploration (MTA) catalogue. Orange triangles are the station network. Maps in a and b have been generated using GMT (v6.6.0, https://github.com/GenericMappingTools/gmt/releases).

We observe a general division along the EAFZ, with high absorption to the north and low absorption to the south, consistent with a similar division in scattering (Fig. 3, and **Figure S3** at 12 Hz). This consistency supports the stability of the technique, even when using different datasets.

The strongest absorption anomaly is observed at the TJ, whereas a constant absorption anomaly lies to the west of the Cardak-Sürgü fault. The high absorption in the TJ is primarily observed during the pre-sequence, due to insufficient coverage during the sequence.

The larger change between pre-sequence and sequence occurs along the Malatya fault, where we observe a transition from high to low absorption, displaying an opposing trend to the scattering behaviour in Fig. 2a, b.

**Fig. 4:**
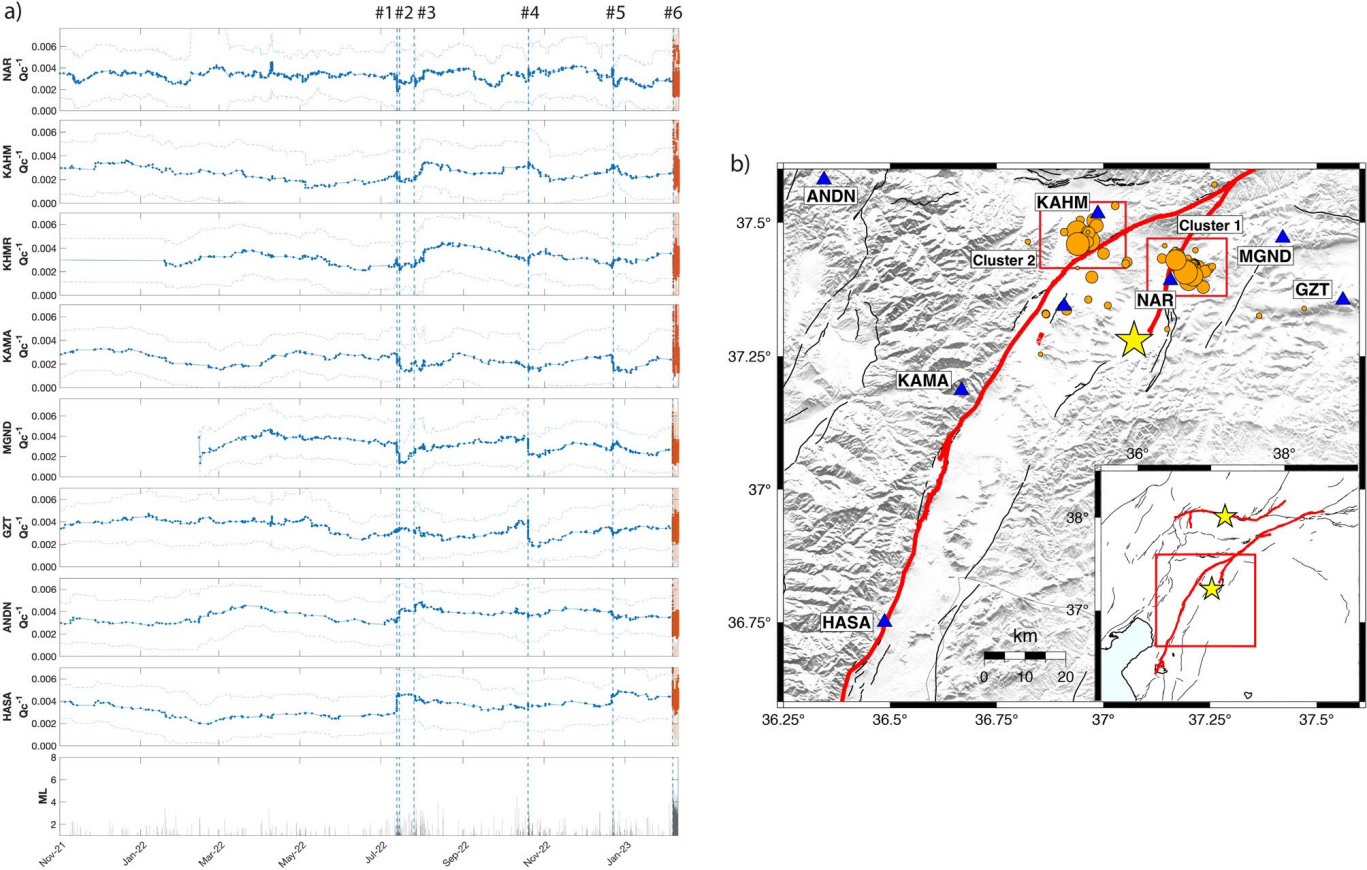
**a**) Timelines of the averaged Qc^− 1^ calculated at 1.5 Hz from the waveforms recorded by eight stations between 01 November 2020 and 10 February 2023. Blue dots represent the moving mean, together with the moving standard deviation. The numbered events are reported in Table 1. The time series at the bottom shows the magnitude of the dataset. **b)** Map of the stations used for the Qc^− 1^ variation in time, and the seismic clusters of July and October 2022. Thick red lines delineate the fault segments that were activated during the 2023 Kahramanmaraş earthquake sequence, and yellow stars are the locations of the Mw 7.8 and 7.5 mainshocks of the 2023 seismic sequences. Black lines are the fault from the Mineral Research and Exploration (MTA) catalogue. Timelines were constructed using MATLAB (vR2024b, https://it.mathworks.com/downloads/). Map b has been generated using GMT (v6.6.0, https://github.com/GenericMappingTools/gmt/releases/).

We further focused on the temporal evolution of attenuation in the preparatory phase of the Mw7.8 mainshock (Fig. 4), following the approach of Gabrielli et al.^19^. The map in Fig. 4b shows the events that have been correlated with the Qc^− 1^ variations, listed also in Table 1. We focused on the period between 01 November 2021 and 10 February 2023, so just a few days after the beginning of the sequence. We chose this time period because, in the area, only two clusters of events occurred before 6 February 2023, occurred on 15 July and 20 October 2022 (Fig. 4b, Clusters 1 and 2).

We detect a clear change in absorption in correspondence with the mainshock of the 6th February (orange dots in Fig. 4), exceeding 60% of the mean value of Qc^− 1^ in all the stations located around the mainshock (KHAM, NAR, KAMA, MDNG), and decreasing to less than 50% moving to farther stations (GZT, ANDN, HASA) (**Figure S4**).

However, the most interesting elements are the variations associated with events preceding the mainshocks. We can indeed observe a Qc^− 1^ change during the events of 13 and 15 July 2022 (Mw3.5 and 3.8), 26 July 2022 (ML 4.4), and 20 October 2022 (ML 4.3), the only clusters that occurred in the area before the 2023 sequence. In general, all these events are witnessing a sudden drop of Qc^− 1^ of −40% and − 50% (Figs. 4-S4) at almost all stations, besides HASA (southern station, with an increment of attenuation of 40%). Moreover, before 13 July, we do not detect a strong variation in attenuation.

**Table 1:** List of events marked by dotted lines in Fig. 4.

Event Date	Time	Lat	Lon	Depth (km)	Magnitude	ID Event
13/07/22	18:15	37.41	37.21	9.15	3.11 Ml	1 - Cluster 1
15/07/22	12:13	37.40	37.21	9.44	3.6 Ml	2 - Cluster 1
26/07/22	06:10	37.48	36.94	7.09	3.97 Ml	3 - Cluster 2
20/10/22	11:34	37.40	37.20	12.7	4 Ml	4 - Cluster 1
23/12/22	17:23	37.43	37.17	8.94	3.5 Ml	5 - Cluster 1
06/02/23	01:28	37.26	36.93	13.72	5.57 Ml	6

We calculated the frequency dependence of the average coda Q (Q_c_) for both periods, fitting a power-law relation *Q(f) = Q*_*0*_*f*^*n*^. The results indicate stable regional properties, with Q_cPre_= 198 ± 69*f*^0.62±0.16^ and Q_cSeq_=187 ± 68*f*^0.58±0.17^. Both datasets show a consistent increase in Q_c_ with frequency (Table 2). Comparing these values with previous studies in the region reveals methodological differences. Our Q_0_ estimates are higher than those reported by Fu et al.^28^, who found a total quality factor Q_tot_ = 145*f*^0.96^ using a non-parametric GIT approach, and by Izgi et al.^23^, who obtained Q_tot_ = 74 *f*^0.7^ for the Central Anatolian Fault Zone. These variations likely reflect the different sensitivity of Coda Q versus direct wave attenuation methods, as well as regional heterogeneity.

**Table 2:** Values of Qc for the Pre-Sequence and 2023 Sequence for the frequency bands of 1.5, 3, 6, and 12 Hz.

Frequency Band	Pre-Sequence	2023 Sequence
1.5 Hz	217 ± 96	204 ± 71
3 Hz	384 ± 122	344 ± 128
6 Hz	666 ± 341	588 ± 230
12 Hz	909 ± 213	769 ± 220

## Discussion

Our results provide the first spatiotemporally resolved separation of scattering and intrinsic attenuation across the East Anatolian Fault Zone; previous attenuation studies in the region were largely static or did not explicitly distinguish these two components. Overall, we detect no major changes in scattering and absorption behaviour across space; however, these two parameters offer complementary insights into the fault and seismic sequence evolution.

Indeed, we observe that scattering increases along the a-Sürgü fault during the sequence (Fig. 2b), directly mapping the increment in fracturing induced by the Mw 7.5 rupture. This association between active faulting and high peak delay is consistent with observations at both sample^29^ and local^20^ scales.

The Cardak-Sürgü fault is a tectonic structure that brings into contact the metamorphic Central Anatolian Crystalline complex with the carbonates and volcanic rocks of the Arabian Plate, thereby creating a rheological contrast (Fig. 2c), which may have led to different fracturing behaviour during the M_w_7.5 event. Our findings further validate the efficacy of this technique in delineating tectonic and geological structures^16,]^^19,]^^29,]^^30^, highlighting its potential for investigating regions with limited tectonic constraints.

The Pütürge area is characterized by high scattering both before and during the sequence; in fact, the pre-sequence dataset al.so comprises data from the January 2020 seismic sequence, with the epicentre in Elazığ^31^. Persistent high scattering following major seismic events has also been observed in other tectonic regions. For instance, the impact of the Mw6.3 L’Aquila earthquake in 2009 on scattering remained evident during the 2016–2017 seismic sequence in the Central Apennines^16^^[,20^.

Moreover, the intersection between the Pazarcik and Pütürge faults is a consistent contrast of low and high scattering (Fig. 2a). This intersection brings into contact the distinct domains of the East Anatolian Plateau and the Arabian Plate, which also corresponds here to a different rheological response, as seen in other tectonic and volcanic zones^20,]^^32,]^^33^. This difference is also supported by the differences between the focal mechanisms of earthquakes generated within the Pütürge/Elâzığ zone and the Pazarcık, found by Billi et al.^34^. Both the Elâzığ and Pazarcık mainshocks are characterized by strike-slip mechanisms. However, their aftershock sequences differ: Elâzığ aftershocks remain strike-slip, whereas Pazarcık aftershocks exhibit mixed behavior^34^ (e.g., normal faulting with minor strike-slip component).

Also, studies of slip distribution along the Pütürge segment revealed that its locked western part did not rupture during the 2020 Elâzığ events^8^^[,35^, which may accumulate substantial strain in what can be described as a seismic gap of the EAFZ (Fig. 2c, red circle). This conclusion is mainly supported by the fact that the southwestern part of the Pütürge fault exhibits a strong heterogeneity contrast, which well aligns with the segmentation described in Cheloni et al.^35^, where this southwestern termination of the Pütürge fault represents a major structural boundary. In their results, this 40–50 km section remained unruptured during both the 2020 and 2023 sequences, and their modeling of interseismic GNSS data confirms that this portion of the fault is strongly locked at depths of 15–20 km. Moreover, the geodetic moment accumulation in this gap since 1905 corresponds to a potential event of Mw 6.6–7.1, and high strain-rate values, observed specifically along this segment, indicating a rapid accumulation of tectonic stress. The comparison of these geodetic findings with our scattering analysis and geology of the area suggests that this area warrants close seismic monitoring due to its high seismic risk.

To the northeast of the map, the Triple Junction (TJ - Figs. 2 and 3) exhibits constant high scattering and absorption, which may be due to the dense fault network in the area and the presence of volcanic rocks. This area has been defined as highly heterogeneous compared with the Kahramanmaraş zone^25^; this is consistent with our results, as we detect similarly high scattering anomalies in the TJ and lower scattering in the Kahramanmaraş area^25^.

The highest absorption anomaly of the TJ zone is in agreement with a low Qc anomaly (high attenuation) at 1 Hz found by Aydin et al.^36^, reflecting the intense tectonic activity^37^, the Neogene-Quaternary magmatic setting, and the thermal fluid circulation^38^, suggested also by the high Vp/Vs^39^.

A similar division in absorption anomalies along the EAF is reported by Izgi et al.^23^. It is important to note that their southern part of the Central Anatolian Fault partially overlaps with our study area. In their Q_i_^−1^ imaging at 1.5 Hz, there is a contrast between higher and lower absorption following a similar trend to ours, with a lower anomaly in the Adana basin, as we record during the sequence^23^. This variation can be associated with significant crustal thinning between the Taurus Mountains (> 40 km thick) and the Adana basin (~ 30 km thick)^40^.

The time series in Fig. 4 provides additional insights into attenuation that are not visible in the map in Fig. 3. A sharp, impulsive change in attenuation (ranging from − 60% to + 60%, **Figure S4**) is clearly observable near the February 6th mainshock. This effect decreases with distance from the faults. For example, station HASA, 70 km from the event, shows variations ranging from − 20% to 50%. The effect of Cluster 1 is still visible (**Figure S5**), even if with much less impact, at stations located above the Cardak-Sürgü zone (> 100 km distance), in a different geological area (Fig. 2c). Notably, station AKCD (**Figure S5**) also recorded the effects of the Mw 5.3 earthquake (April 9, 2022) that occurred in the Elazığ zone. The temporal variation reported by Gabrielli et al.^19^ of Qc^− 1^ in the Central Apennines during the 2016 seismic sequence showed opposite variations at two stations during the sequence, attributable to fluid migration within the fault network. Here, the general behaviour is to have a drop, a decrease in attenuation, at each event of 2022, at all stations, besides HASA and ANDN, both at around 60–70 km from the cluster areas. Coseismic drops of seismic velocity^41^ revealed higher drops near the fault rupture during the seismic event of the 06/02/2023 (e.g., KAMA dv/v = 0.53 at 1.5 Hz), rather than far from it (e.g., HASA dv/v = 0.13 at 1.5 Hz). While in the Central Apennines, this opposite behaviour has been attributed to the migrations of fluid during the sequence^19^, the current sequence is not considered to be fluid driven. The anomaly at station HASA is likely due to its location at the margin of the Amik basin. This setting suggests a higher absorption caused by sedimentary deposits, an effect well-documented in other basin areas^15^^[,16^.

Our time-dependent analysis of Qc^− 1^ detects significant changes in the crustal properties during the preparatory phase, associated with moderate pre-sequence seismicity, months before the February 6th mainshocks. This aligns well with other studies on the preparatory process: Picozzi et al.^42^ identified an activation stage in June 2002 through energy variations, while Núñez-Jara et al.^43^ observed a decreasing *b-*value eight months before the sequence, along with cluster localizations, suggesting a progressive weakening in the Narlı fault segment and an increment of stress.

In this context, the drop in absorption we observe during the 2022 clusters (July and October, the same one analysed by Núñez-Jara et al.^43^) provides a physical signature of rheological variations and crustal modification, reflecting a change in the rheological status and characteristics of the medium in the fault zone. These observations of Qc^− 1^ in the pre-sequence highlight the capability of attenuation to detect preparatory processes in tectonic settings and track rheological evolutions of active fault systems.

Finally, these results highlight the importance of continuous, multi-parameter monitoring of active fault zones. Because attenuation is sensitive to both fracturing and fluid presence, its temporal variations can provide key insights into the evolving state of fault systems. Integrating long-term attenuation monitoring with velocity tomography, microseismicity, and geodetic data can improve our understanding of the physical processes that precede and follow major earthquakes.

The combined analysis of scattering and absorption is particularly valuable in capturing the interplay between lithology, fault damage, and fluid migration. Attenuation imaging and its time-series thus emerge as a powerful tool for investigating fault-zone complexity and contribute meaningfully to both seismic-hazard assessment and the broader understanding of earthquake physics.

## Methods

### Peak delay -scattering

The peak delay method involves measuring the lag time between the S-wave arrival and the maximum amplitude of the envelope of each waveform, providing quantitative information about multiple scattering in the crust^30^.

**Fig. 5:**
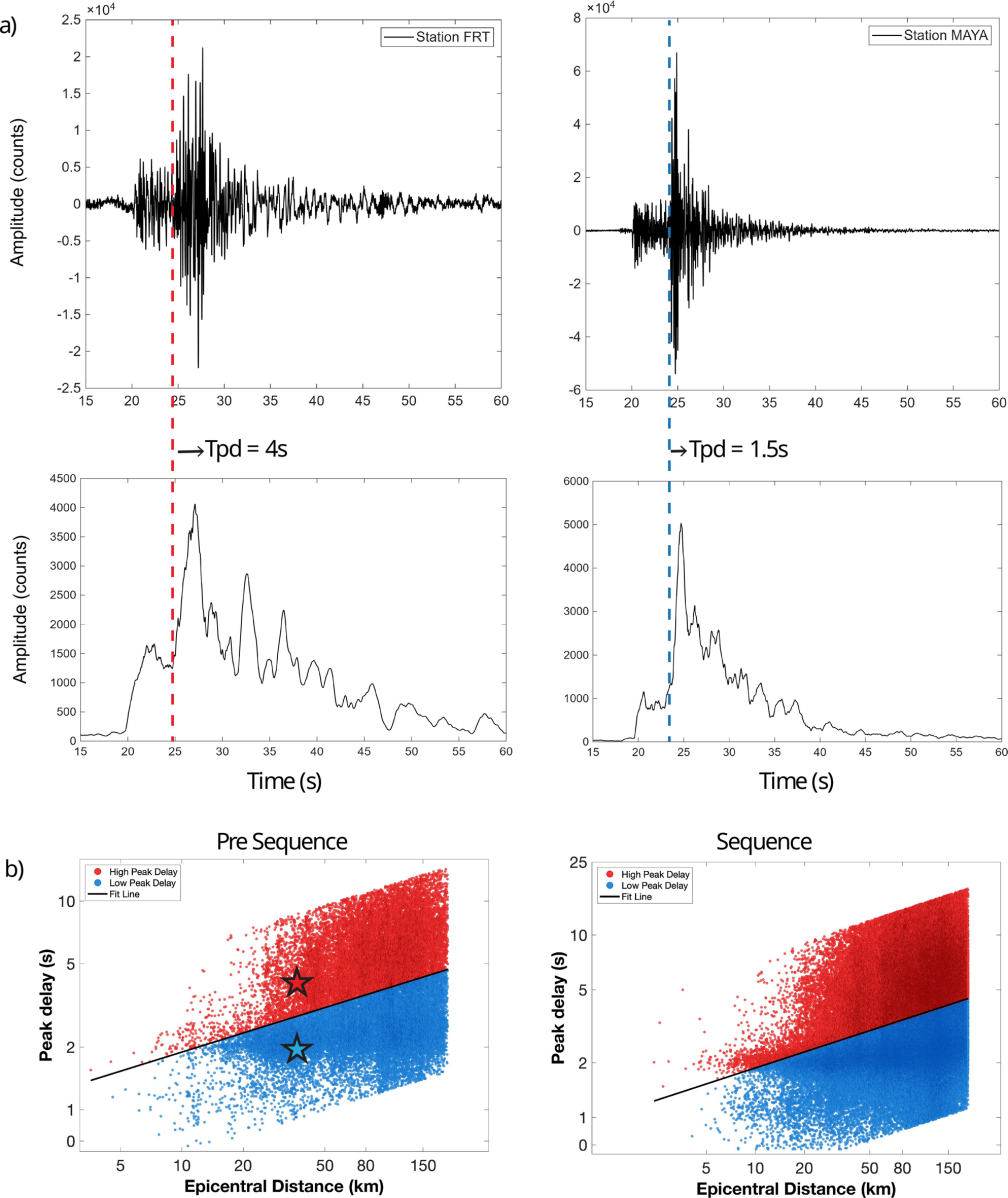
**a**) Example of an event recorded at NARI and MAYA stations at the same epicentral distances, 40 km (top and middle panels), and their envelopes at 1.5 Hz (bottom panels). The vertical blue and red dashed lines display the S-wave onset, and the black arrow shows the peak delay time. **b**) Log–log plot of peak delay (s) as a function of the distances (km) for the Pre-sequence and the 2023 Sequence at 1–5 Hz. The top red area describes high scattering, the blue area the low scattering. Red and Blue stars represent the position of the events in the top panel a). Plots have been created using MATLAB (vR2024b, https://it.mathworks.com/downloads/).

Seismograms have been filtered with a band-pass Butterworth filter fourth order, in the central frequency of 1.5 Hz (1–2 Hz frequency band) (Fig. 5a).

After calculating the peak delay for each waveform, we plot it vs. the epicentral distance, and we find the best-fit line (theoretical peak delay - (Fig. 5b). We then compute the difference between the measured peak delay time and the theoretical peak delay:

1 $$\:\varDelta\:{log}_{10}t\left(f\right)\:={log}_{10}{t}_{i}^{PD}\left(f\right)\:-\:{log}_{10}{t}_{}^{PD}\left(f\right)\:$$

with $\:\:{t}_{}^{PD}\left(f\right)\:=\:A\left(f\right)+B\left(f\right)\:\cdot\:\:{log}_{10}R$, with A(f) and B(f) being the regression coefficients and R the epicentral distance (Fig. 5b).

After obtaining $\:\varDelta\:{log}_{10}t\left(f\right)$, we allocate to each block of the grid the weighted average of all rays crossing it, obtaining positive (red cells) and negative (blue cells) values, which indicate high and low scattering, respectively. As the regionalization approach does not consent to having a resolution test as a checkerboard map, we use the common technique of the hit count/ray density map^19^^[,30^, taking into consideration only the cells crossed by more than 10 rays (Fig. 1b), following the approach of Calvet et al.^30^ and Gabrielli et al.^29^.

Here, we added two additional tests. The first one, a resampling test (**Figure S6a**), where we randomly selected a subset of the Sequence data to match the pre-sequence ray density (20722 rays), to verify if the denser ray coverage introduced artifacts. We had to adopt an intersection of the hit count distribution of the two sequences to ensure that we are comparing the same physical volumes under identical sampling conditions. Although we observe small changes at the edges of the maps, the test results show a good agreement with our results, and therefore a good stability.

We further performed a second test, a bootstrap test, where we generated 100 independent realizations of the maps by randomly selecting 85% of the dataset in each iteration, and then estimated their standard deviation (**Figure S7**). The standard deviations for both sequences show low values across the entire study area (max 0.022), with the highest stability observed in the central region, where the main anomalies are discussed. We also provide the mean map, derived from all 100 iterations, which shows a really strong stability in terms of the anomaly placement, if compared with our results. This is a further confirmation that the anomalies are not artifacts of ray-path distribution or specific subsets of data.

### Coda Attenuation

Coda waves are the train waves after the S-wave arrival and give us information about scattering and absorption properties in the medium (e.g., presence of fluids and heterogeneities).

For the late coda, we can assume that the value of Qc can be a description of absorption (intrinsic attenuation) Qi^44^.

We measure the decay rate of the coda envelope by quantifying the inverse of the coda quality factor Qc^− 1^, as follows^11^:

2 $$\:E(t,f)\:=\:S\left(f\right){{t}^{-\alpha\:}exp\left(\frac{-2\pi\:ft}{{Q}_{c}}\right)}_{}$$

where *S(f)* includes source and site terms, and α is equal to 3/2 in the asymptotic case of diffusion^12^.

We compute Qc^− 1^, by linearizing Eq. 2:

3 $$\:\frac{ln\left[E\right(t,f)\cdot\:{t}^{\alpha\:}]}{2\pi\:f}\:=\:\frac{ln\left[S\right(f\left)\right]}{2\pi\:f}\:-\:\frac{1}{{Q}_{c}}t$$

**Fig. 6:**
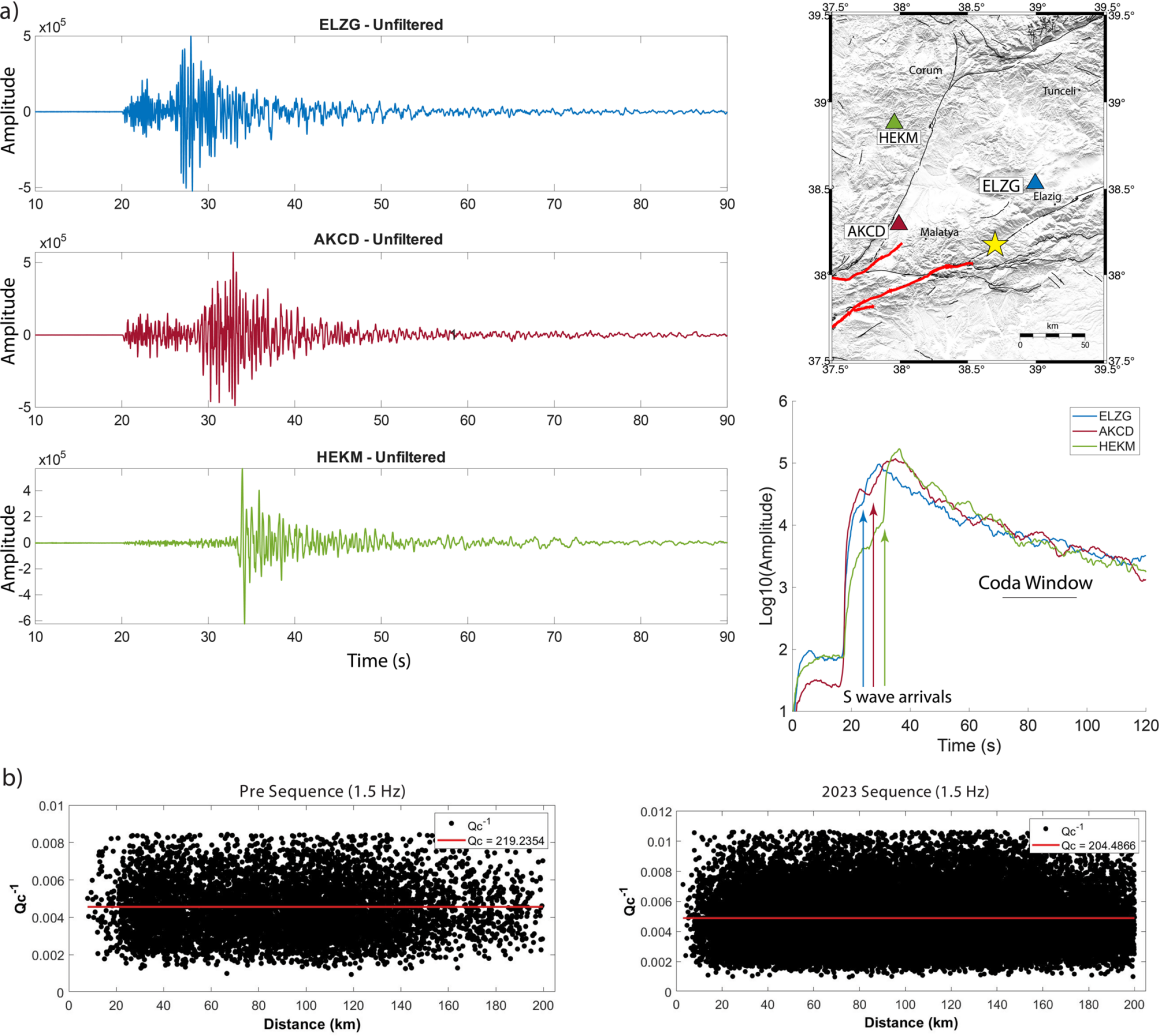
**a**) Example of unfiltered seismograms and their envelopes at 1.5 Hz at stations ELZG, AKCD, and HEKM for the event of 23/02/2023 Ml 3.6 (map on the top right). Coda window is set at 2ts from the S wave arrival, with a fixed length of 20 s; **b**) Qc^− 1^ as a function of epicentral distance at 1.5 Hz for the Pre-Sequence and the 2023 Sequence. Black dots are for each waveform obtained from the linear approach approximation in Eq. 2. The red line represents the average Qc^− 1^. Plots have been created using MATLAB (vR2024b, https://it.mathworks.com/downloads/) and GMT (v6.6.0, https://github.com/GenericMappingTools/gmt/releases/).

For the analysis, the coda window starts at 2 times the S wave arrival, and with a length of 20 s (Fig. 6a). These choices were made after demonstrating the stability of Qc^− 1^ at distances between 5 and 200 km (Fig. 6b), a necessary condition to fulfil the assumption of Qc^−1^≈Qi^[− 1 44^. It is indeed necessary that coda waves enter the diffusive regime at late lapse times. To further assess the validity of the diffusive approximation in our study area, we calculated the Mean free time (MFT). We determined the MFT using the scattering quality factor Q_sc_ previously calculated by Izgi et al. (2023) for the same region. At the reference frequency of 1.5 Hz, they report a Q^−1^_sc_≈ 0.004 (Q_sc_ ≈ 250). Using the scattering coefficient formula ηs = 2πf/VsQ_sc_, and assuming Vs = 3.5 km/s, we obtain ηs of 0.0107 km-1. The Mean Free Path (MFP), defined as 1/ηs, leads to a Mean Free Time (MFT = MFP/Vs) of ~ 26.7 s. In our dataset, the average S-wave arrival time (t_s_) is 36.65 s for the pre-sequence and 35.92s for the Sequence. Since our coda window starts at 2ts, the corresponding lapse time (t_lapse_ ≈ 72–73 s) is significantly greater than the MFT. Under the condition t_lapse_ > MFT, the seismic energy can be considered to have entered the diffusive regime.

For the regionalization, following the approach used by Gabrielli et al.^45^, we mapped the spatial variation using the kernel functions of the diffusive regime by Del Pezzo et al.^46^. We then performed, as for the peak delay, the weighted average of the value of Qc^− 1^ for each cell block, when crossed by more than 10 rays (Fig. 1b). To further assess the robustness of the spatial variation of attenuation, together with the hit count map of Fig. 1b, we applied the technique developed by Del Pezzo and Ibáñez^47^ to show the spatial distribution of the normalized standard deviation σ associated with the single Qc values. In this approach, a resolution is defined as the inverse of the normalized standard deviation of the weighted average, calculated for each grid cell (**Figure S8**).

We determined the weights by the numerical value of the kernel function at the centre of each cell. Higher resolution values indicate more reliable estimates in each cell, and vice versa. Gabrielli et al.^45^, Del Pezzo and Ibáñez^47^, and Castro-Melgar et al.^48^ applied this new representation of uncertainty in volcanic and tectonic zones. This type of test is the most suitable for the projection mapping of the Q_c_ weighting function that we performed.

As for the peak delay, we performed the resampling (**Figure S6b**) and bootstrap tests (**Figure S9**) also for the Qc^− 1^ imaging. Also in this case, we observe a strong stability of the anomalies, allowing us a confident interpretation of the results.

For Fig. 4 and **Figure S5**, we calculated the moving average, with moving windows having a length of 30 events, and moving them one event at a time. We also plotted the standard error, the light blue line above and below the moving average, in Fig. 4 and **Figure S5**.

## Electronic Supplementary Material

Below is the link to the electronic supplementary material.

Supplementary Material 1

## Data Availability

Output files are available in the Zenodo repository, [https://zenodo.org/uploads/18031669](https:/zenodo.org/uploads/18031669).

## Code availability

The workspace used to perform analyses with the open-access MuRAT2D MATLAB© code is available at https://github.com/LucaDeSiena/MuRAT2D.
