# Supplementary material for: Seismic attenuation transients reveal progressive crustal modification before and during the 2023 Türkiye earthquakes
Source: Sci Rep. 2026 May 15;16:22166. doi: 10.1038/s41598-026-52463-1 (PMC13370003; doi:10.1038/s41598-026-52463-1)
Supplement: Supplementary file 1 — Supplementary Material 1 [file 41598_2026_52463_MOESM1_ESM.docx]

Seismic attenuation transients reveal progressive crustal modification before and during the 2023 Türkiye earthquakes

**Simona Gabrielli^1*^, Aybige Akinci^1^, Yijian Zhou^2^, Edoardo Del Pezzo^3,4^, Luca De Siena^5^**

1. Istituto Nazionale di Geofisica e Vulcanologia, Rome, Italy
2. Caltech GPS, Pasadena, California, US
3. Osservatorio Vesuviano, Istituto Nazionale di Geofisica e Vulcanologia, Napoli, Italy
4. Istituto Andaluz de Geofisica, Universidad de Granada, Granada, Spain
5. Dipartimento di Fisica e Astronomia “Augusto Righi”, Alma Mater Studiorum, Bologna, Italy

* Corresponding Author: simona.gabrielli@ingv.it

**Supplementary Material**


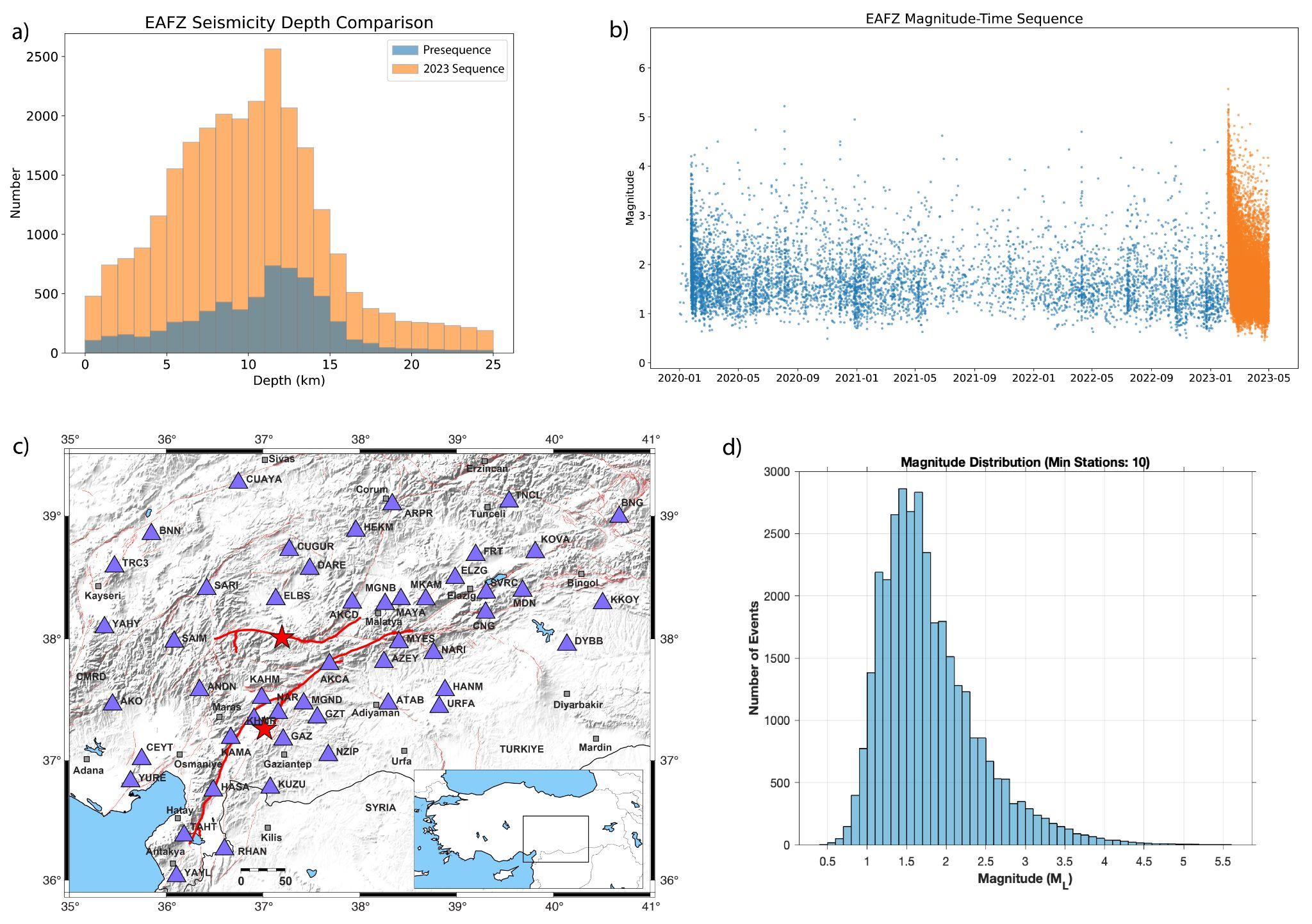


**Figure S1. Statistical information about the dataset and waveforms.** a) Number of events vs depth distribution for the Pre Sequence phase (in blue) and 2023 sequence (orange). b) Plot of magnitude over time (blue color represents the presequence catalog, the orange the 2023 sequence). c) Stations distribution for both datasets. d) Magnitude distribution per events.


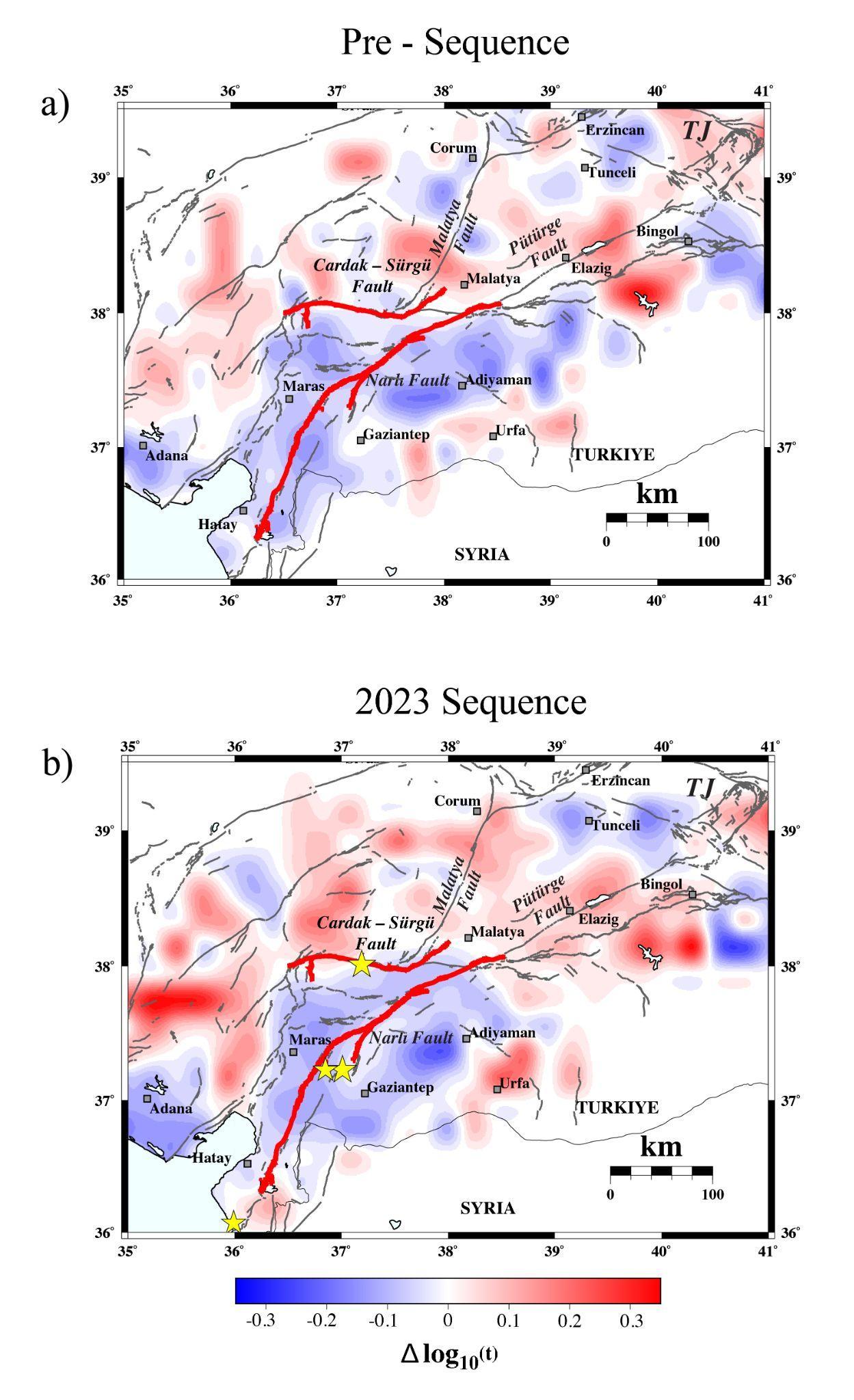


**Figure S2. Spatial and temporal variation of peak delay at *fc* = 12 Hz.** Spatial and temporal variation of peak delay at *fc* = 12 Hz during the a) pre-seismic sequence and b) the 2023 sequence. The maps display the absolute values of the frequency-dependent peak delay, Δlog₁₀t(*f*). Negative values (cold colors, blue) indicate low-scattering attenuation, while positive values (hot colors, red) correspond to high-scattering attenuation. The average Δlog₁₀t(*f*) over the region is approximately zero, serving as a reference.


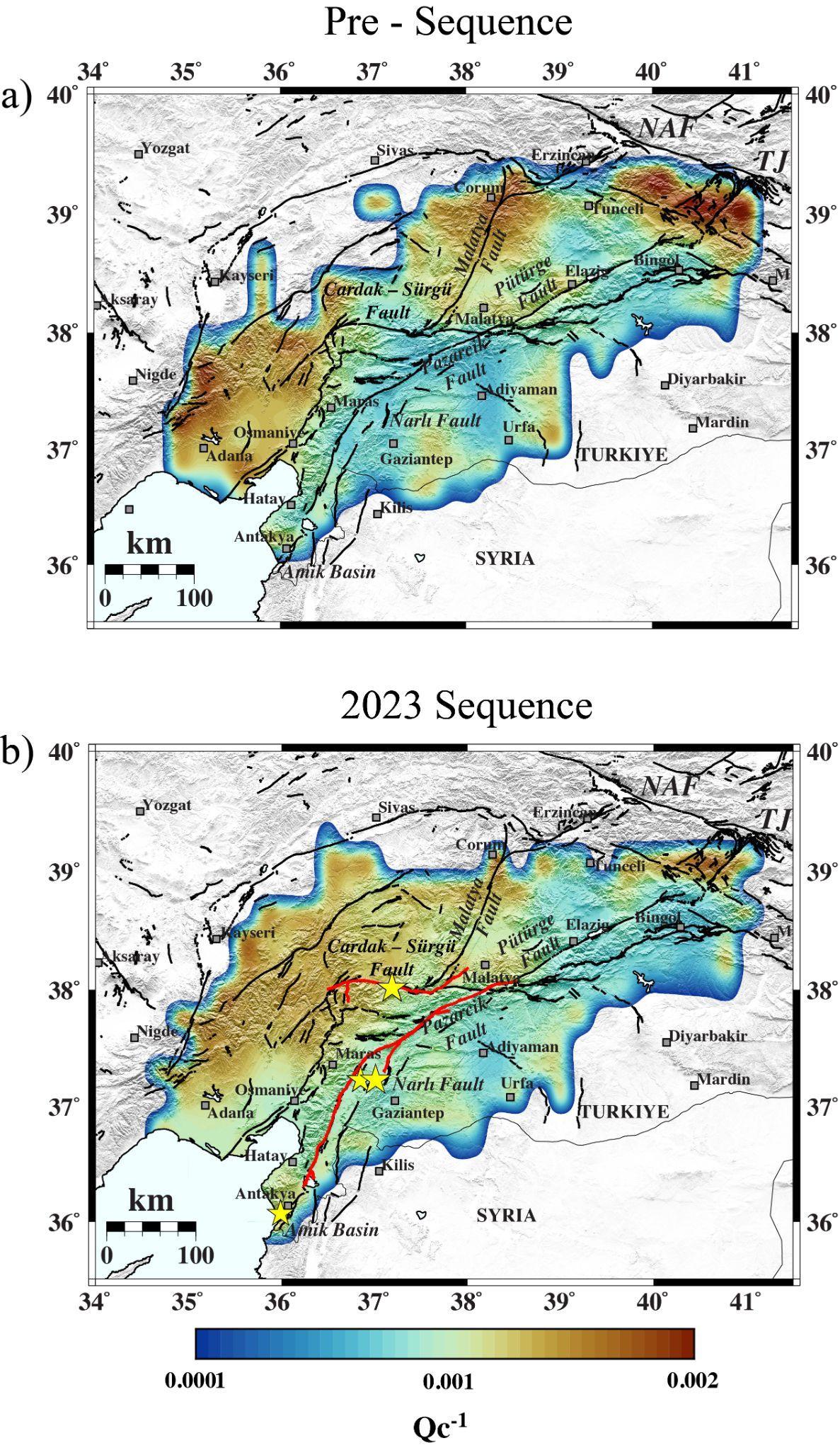


**Figure S3. Spatial and temporal variation of Qc^-1^ at *fc* = 12 Hz.** Spatial and temporal variation of Qc^-1^ at *fc* = 12 Hz during a) the pre-seismic sequence and b) the 2023 sequence.


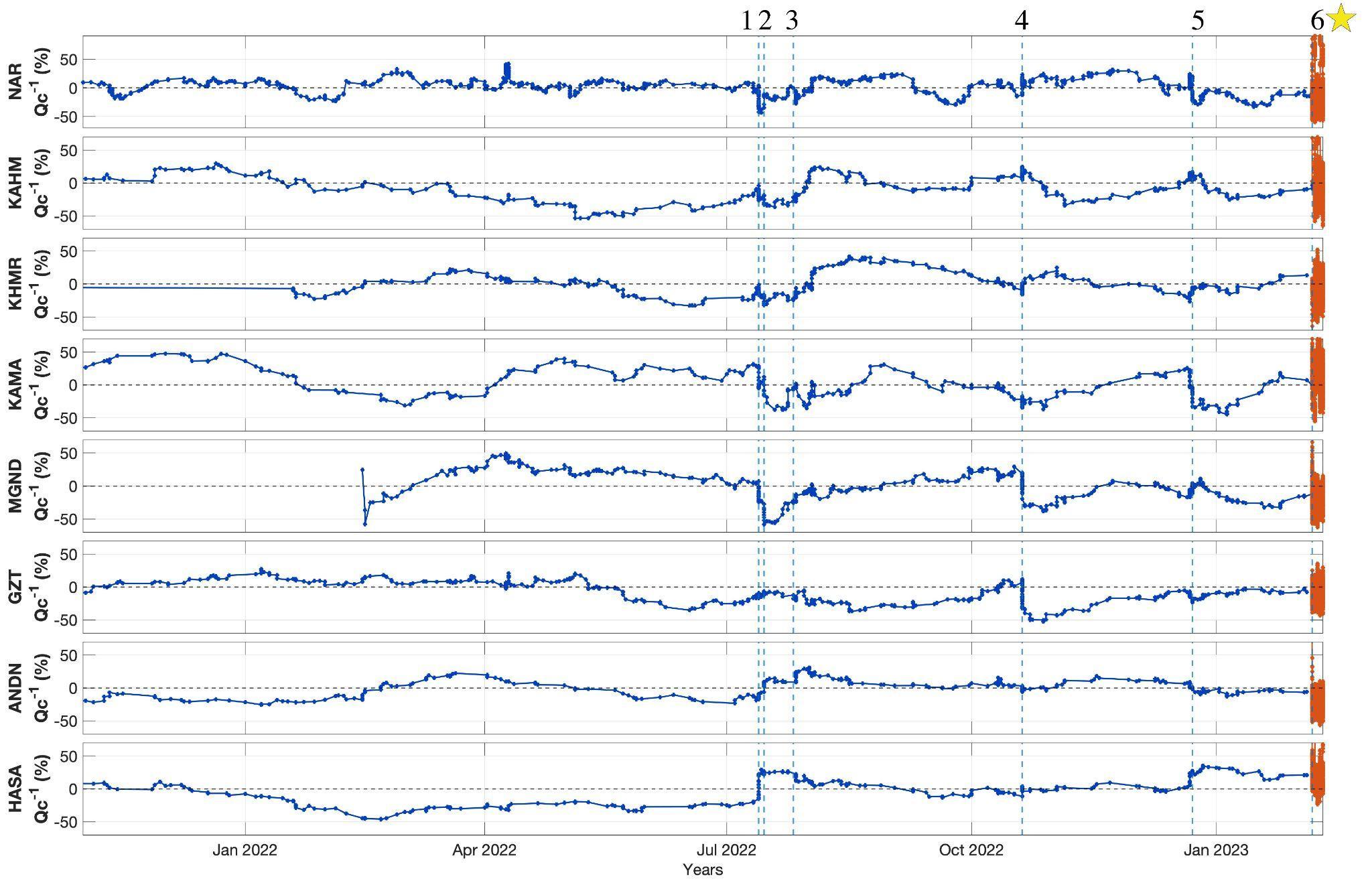


**Figure S4. Percentage difference of Qc^-1^.** Percentage difference of Qc^-1^ for the stations analysed in **Figure 4**. We calculated the difference as ((Moving mean - Average Qc^-1^)/ Average Qc^-1^)*100.


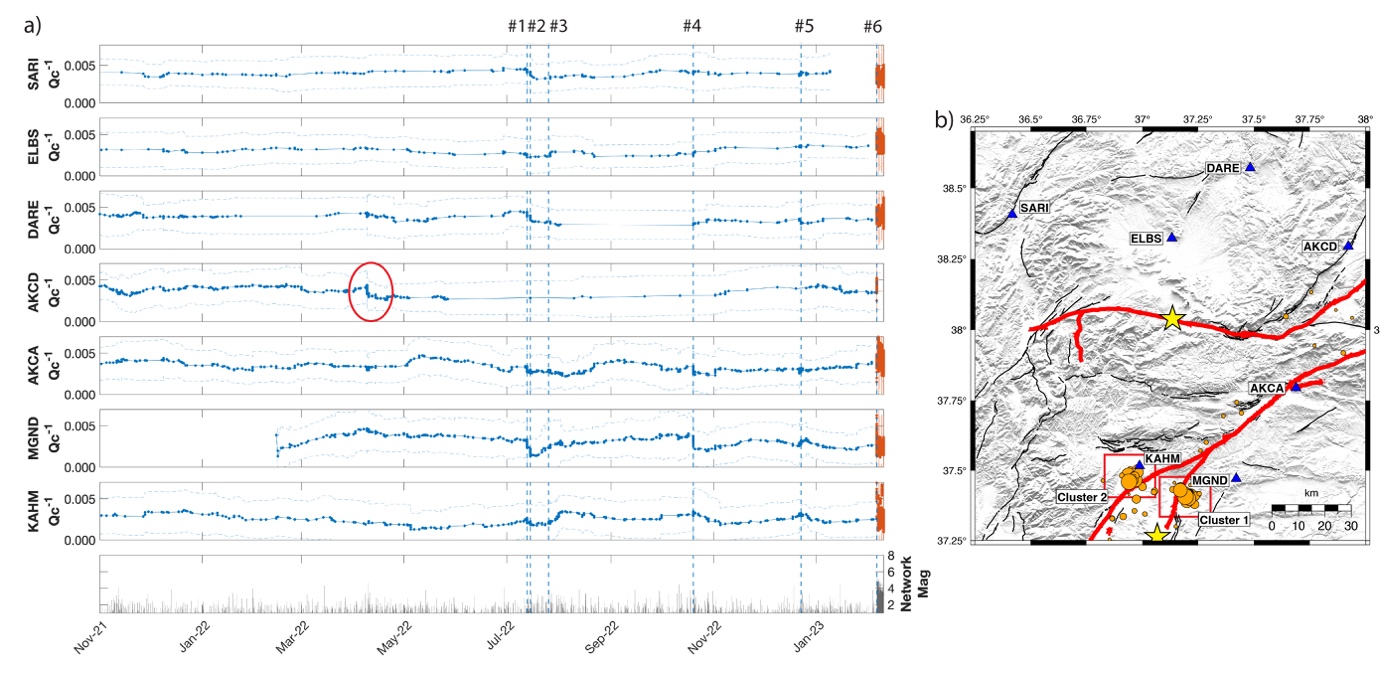


**Figure S5. Timelines of Qc^-1^ in Cardak-Sürgü zone.** **a**) Timelines of the averaged Qc^−1^ calculated at 1.5 Hz from the waveforms recorded by seven stations between 01 November 2020 and 10 February 2023 in the Cardak-Sürgü zone. Blue dots represent the moving mean, together with the moving standard deviation. The numbered events are reported in Table 1 in the main manuscript. The time series at the bottom shows the magnitude of the dataset. The red circle represent the Mw 5.3 earthquake (April 9, 2022) in the Pütürge zone. **b)** Map of the stations used for the Qc^-1^ variation in time, and the seismic clusters of July and October 2022. Thick red lines delineate the fault segments that were activated during the 2023 Kahramanmaraş earthquake sequence, and yellow stars are the locations of the Mw 7.8 and 7.5 mainshocks of the 2023 seismic sequences. Black lines are the fault from the Mineral Research and Exploration (MTA) catalogue.


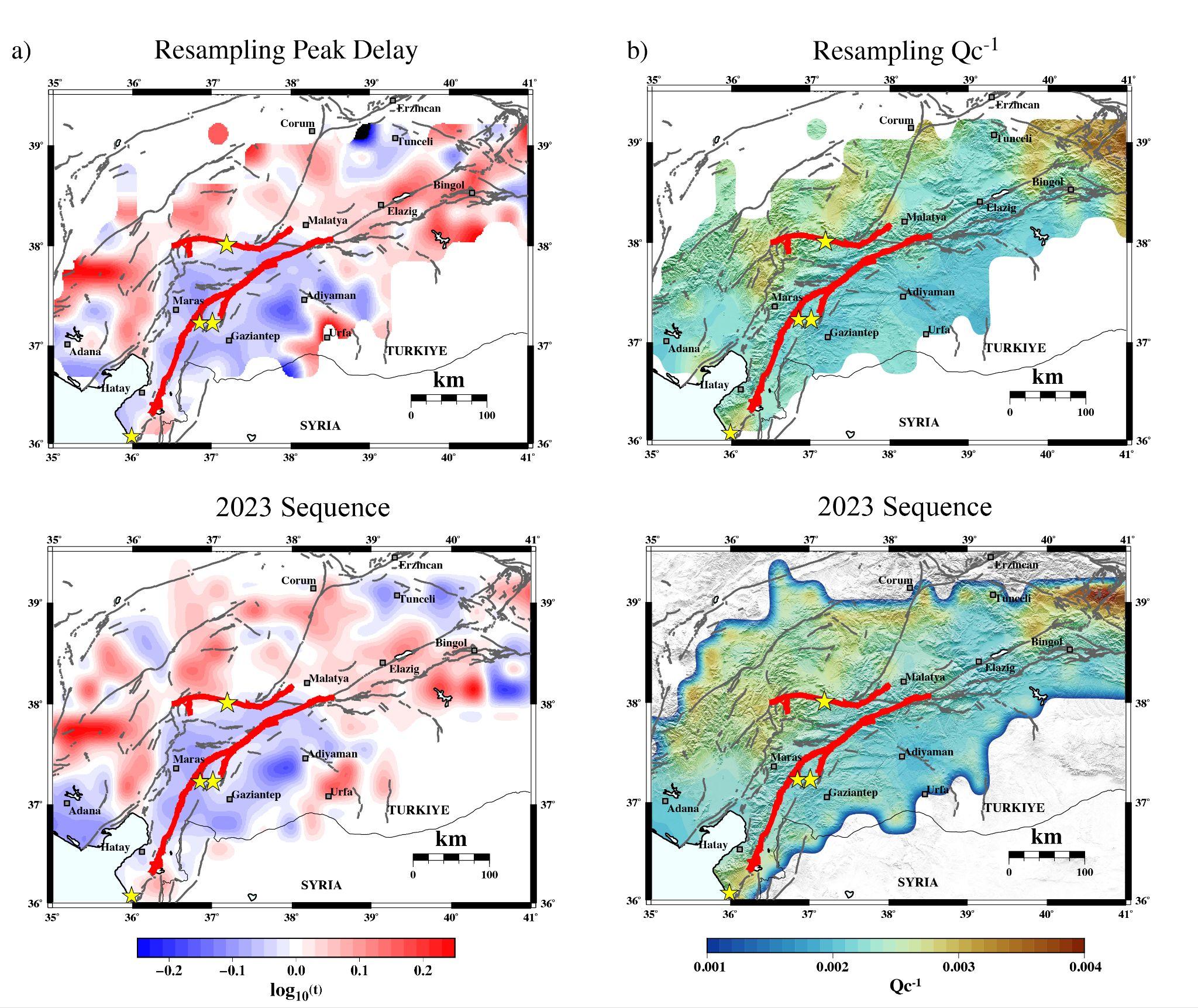


**Figure S6.** **Resampling of the Sequence phase.** Resampling of the sequence for a) the peak delay spatial distribution and b) for the Qc^-1^, by using the same number of rays of the Pre-Sequence phase (20722) and randomly selecting a subset of the Sequence data.


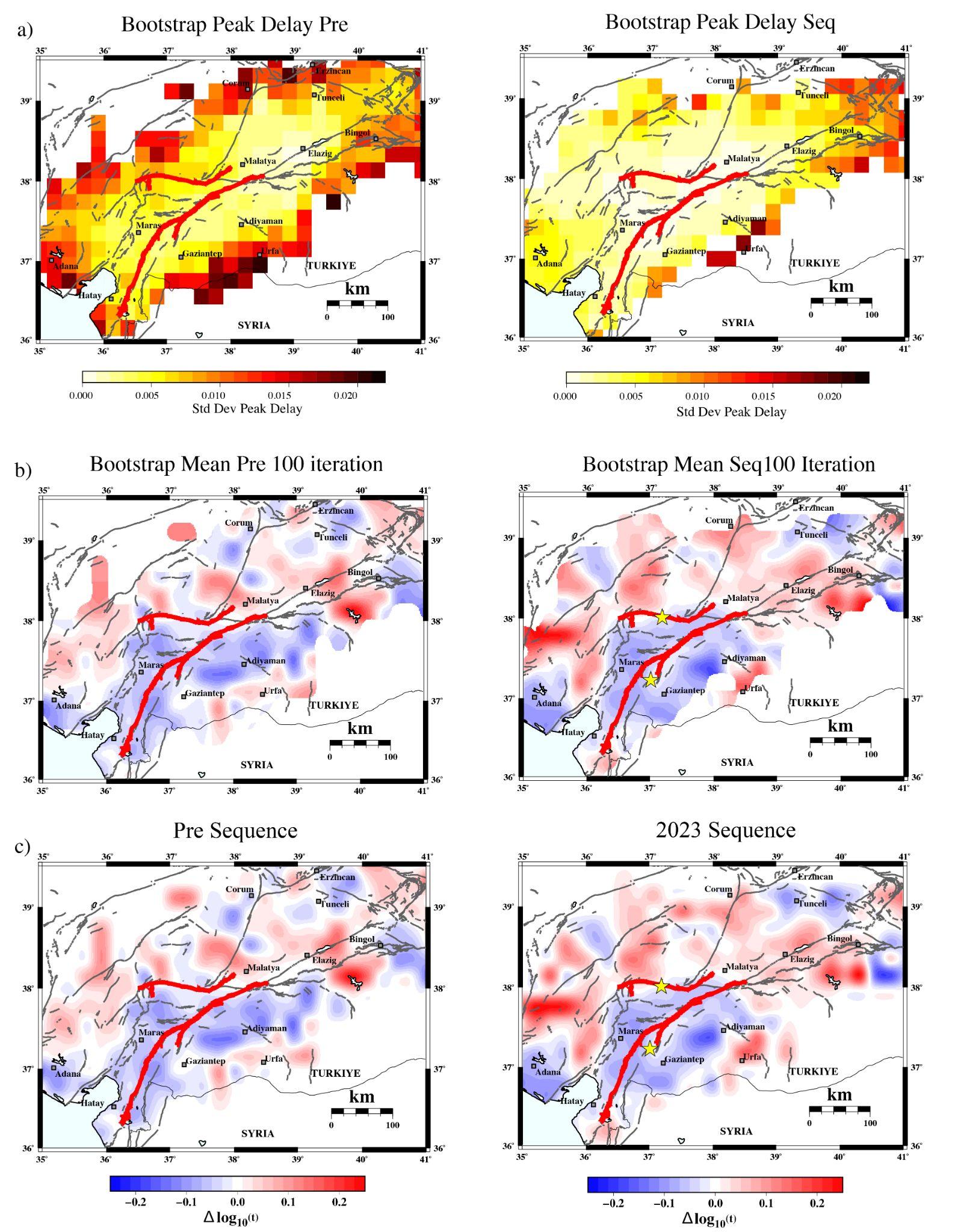


**Figure S7.** **Bootstrap for peak delay. a)** standard deviation of the 100 independent realizations of the maps by randomly selecting 85% of the dataset in each iteration; **b)** plot of the mean of all the 100 iterations; **c)** peak delay results of Figure 2 for comparison with the bootstrap test.


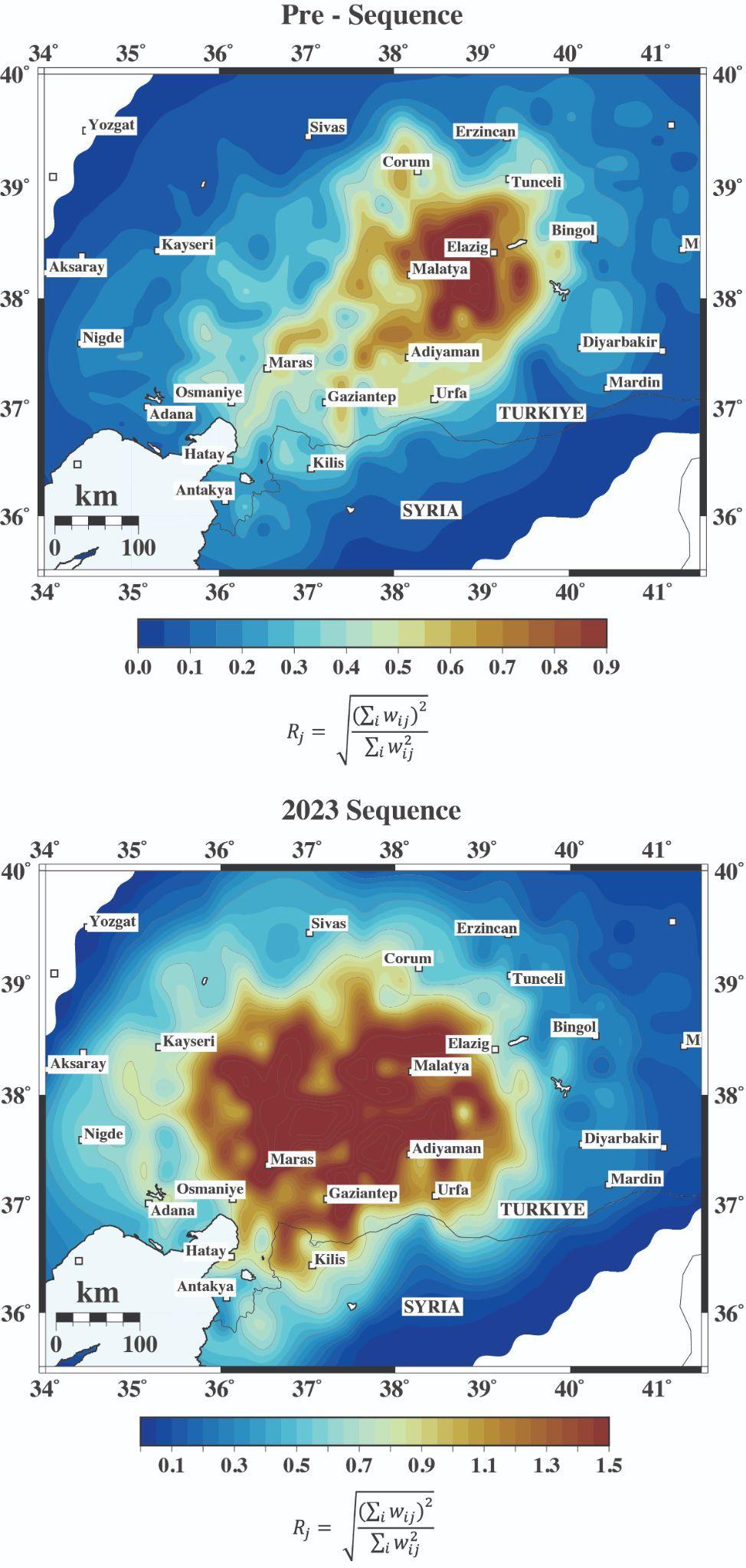


**Figure S8.** **Resolution test for Qc using standard deviation distribution.** Resolution test of the spatial distribution of the standard deviation σ for the Qc^-1^ for the Pre-sequence and Sequence phase


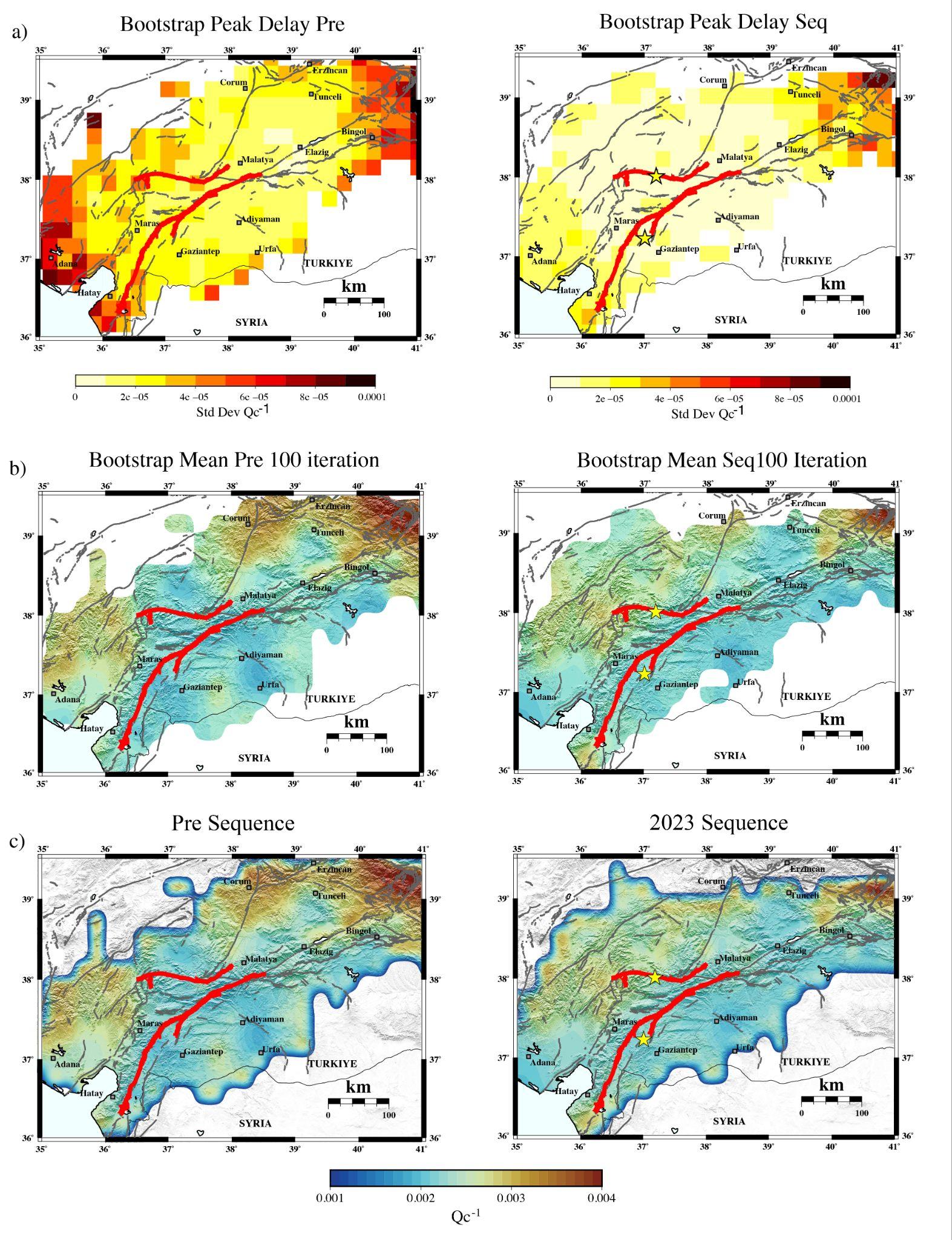


**Figure S9.** **Bootstrap for Qc^-1^. a)** standard deviation of the 100 independent realizations of the maps by randomly selecting 85% of the dataset in each iteration; **b)** plot of the mean of all the 100 iterations; **c)** Qc^-1^ results of Figure 3 for comparison with the bootstrap test.
